# Noninvasive Evaluation of the Pathologic Grade of Hepatocellular Carcinoma Using MCF-3DCNN: A Pilot Study

**DOI:** 10.1155/2019/9783106

**Published:** 2019-04-28

**Authors:** Da-wei Yang, Xi-bin Jia, Yu-jie Xiao, Xiao-pei Wang, Zhen-chang Wang, Zheng-han Yang

**Affiliations:** ^1^Department of Radiology, Beijing Friendship Hospital, Capital Medical University, Beijing 100050, China; ^2^Beijing Key Laboratory of Translational Medicine on Liver Cirrhosis, Beijing 100050, China; ^3^Faculty of Information Technology, Beijing University of Technology, Beijing 100124, China

## Abstract

**Purpose:**

To evaluate the diagnostic performance of deep learning with a multichannel fusion three-dimensional convolutional neural network (MCF-3DCNN) in the differentiation of the pathologic grades of hepatocellular carcinoma (HCC) based on dynamic contrast-enhanced magnetic resonance images (DCE-MR images).

**Methods and Materials:**

Fifty-one histologically proven HCCs from 42 consecutive patients from January 2015 to September 2017 were included in this retrospective study. Pathologic examinations revealed nine well-differentiated (WD), 35 moderately differentiated (MD), and seven poorly differentiated (PD) HCCs. DCE-MR images with five phases were collected using a 3.0 Tesla MR scanner. The 4D-tensor representation was employed to organize the collected data in one temporal and three spatial dimensions by referring to the phases and 3D scanning slices of the DCE-MR images. A deep learning diagnosis model with MCF-3DCNN was proposed, and the structure of MCF-3DCNN was determined to approximate clinical diagnosis experience by taking into account the significance of the spatial and temporal information from DCE-MR images. Then, MCF-3DCNN was trained based on well-labeled samples of HCC lesions from real patient cases by experienced radiologists. The accuracy when differentiating the pathologic grades of HCC was calculated, and the performance of MCF-3DCNN in lesion diagnosis was assessed. Additionally, the areas under the receiver operating characteristic curves (AUC) for distinguishing WD, MD, and PD HCCs were calculated.

**Results:**

MCF-3DCNN achieved an average accuracy of 0.7396±0.0104 with regard to totally differentiating the pathologic grade of HCC. MCF-3DCNN also achieved the highest diagnostic performance for discriminating WD HCCs from others, with an average AUC, accuracy, sensitivity, and specificity of 0.96, 91.00%, 96.88%, and 89.62%, respectively.

**Conclusions:**

This study indicates that MCF-3DCNN can be a promising technology for evaluating the pathologic grade of HCC based on DCE-MR images.

## 1. Introduction

Hepatocellular carcinoma (HCC), the most common primary malignant liver tumor, is the second most common cause of death related to malignancy in the world, and more than 500,000 new patients are diagnosed annually [1, 2]. Although the surgical resection of HCC has been improved, patient prognosis remains poor due to the high recurrence rate. According to the “World Health Organization classification of tumors of the digestive system”, HCC can be classified into four pathologic grades, well-differentiated (WD), moderately differentiated (MD), poorly differentiated (PD), and undifferentiated, based on the tumor's cellular and structural atypia. The pathologic grade of HCC is one of the most important factors in evaluating early recurrence after surgical resection [3]. Compared to WD or MD HCCs, PD HCC has a poorer prognosis and higher tumor recurrence. PD HCC is also associated with a worse survival rate than WD or MD HCCs. Therefore, it is helpful to evaluate the pathologic grade of HCC before treatment. Preoperative liver biopsy is the gold standard for pretreatment pathologic grade of HCC; however, this method is not widely used in clinical practice due to several limitations, including invasiveness, sampling error, and bleeding. The development of noninvasive imaging techniques to safely and accurately assess the pathologic grade of HCC would benefit the selection of an optimal treatment method for patients and improve their survival rate.

Increasing numbers of studies have explored noninvasive evaluations of the pathologic grade of HCC, such as diffusion-weighted imaging (DWI) to assess water diffusion and dynamic contrast-enhanced magnetic resonance images to evaluate tumor vascularity [1, 4]. One alternative imaging-based approach to evaluate the pathologic grade of HCC is to assess the internal structure or texture. Texture can be defined as a complex visual pattern in an image that consists of simpler subpatterns with characteristic features [5, 6]. These features can be objectively assessed via quantitative texture analysis. Wu Z et al. [7] evaluated the diagnostic accuracy of texture analysis in determining the malignancy of HCCs based on contrast-enhanced MR images, and they found that both gray-level run-length nonuniformity and average intensity can reflect the pathologic grade of HCC.

In recent years, convolutional neural networks (CNNs) have become one of the most advanced deep learning networks. CNNs exhibit a powerful mechanism in representation learning directly from data instead of manual features while displaying good performance in revealing the local characteristics instead of global characteristics. Deep learning with CNNs has reportedly [8] achieved good performance in the pattern recognition of images/videos. As one of most important areas of computer vision, CNNs are being applied to medical image analysis [9, 10]. Some preliminary achievements of computer-aided diagnostic techniques based on CNNs have also been obtained in medical image analysis for the detection, segmentation, and grading of abdominal lesions in a broad spectrum of diseases [11–14]. Regarding practical clinical experience in the diagnosis of HCC from DCE-MR images, the salient appearance features in MR images and the changes among different phasic images serve as vital clues for determining the grade of HCC. Therefore, we hypothesize that a CNN model, especially 3DCNN, can reveal image characteristics and diagnostic patterns that reflect the pathologic grade of HCC, taking into account both spatial and temporal information.

In this study, we use a multichannel fusion 3D convolutional neural network (MCF-3DCNN) to extract temporal sequence information and spatial texture information from five-phasic DCE-MR images. Based on the learned temporal-spatial features, the grade of HCC is determined at the end of the network of MCF-3DCNN. To develop a well-trained MCF-3DCNN diagnosis model, patient samples were annotated by experienced radiologists. This retrospective study aims to investigate the diagnostic performance of our proposed MCF-3DCNN model for the differentiation of the pathologic grade of HCCs based on DCE-MR images.

## 2. Materials and Methods

### 2.1. Patients

This retrospective study was approved by the Institutional Human Ethics Board after waiving written informed consent. From January 2015 to September 2017, 132 consecutive patients underwent dynamic contrast-enhanced MRI (DCE-MRI) and other conventional magnetic resonance imaging sequences for the evaluation of HCC in the Department of Radiology, Beijing Friendship hospital. The inclusion criteria were as follows: (1) pathologic grade of the HCCs was available; (2) no previous treatment, such as liver resection, transcatheter arterial chemoembolization, radiofrequency ablation, or percutaneous ethanol injection; (3) five-phasic liver DCE-MR images were available, including precontrast, later arterial, portal venous, equilibrium phase, and delay phase images. The exclusion criteria were as follows: (1) prominent artifacts that affected the observation of HCCs; (2) an interval between MRI examination and resection longer than 2 weeks; (3) inaccurate time point of phase. As a result, the study included 42 patients, with 34 men and 8 women. The mean age was 59.35±8.09 years, with a range from 40 to 80 years. The patients' demographics and pathologic information are summarized in Table 1.

### 2.2. Pathologic Examinations

Patient's lesion specimens were obtained from different way including surgical resection, tumor biopsy, and liver transplantation. The decisions to take were made according to a combination of tumor's size, location, number, the presence/absence of metastasis, the status of patient and liver function. Accordingly, two patients with tumors more than two underwent liver transplantation; 12 patients without indication for surgery underwent tumor biopsy; the remaining patients underwent either open liver resection or laparoscopic liver resection.

All obtained specimens were first fixed in 10% neutral-buffered formalin and then embedded in paraffin wax. Specimens embedded in paraffin blocks were cut into four- to five-*μ*m-thick slices in preparation for histochemical staining. Neoplastic lesions were subjected to hematoxylin-eosin staining, and Hepar-1, cytokeratin 19, and c-kit staining were performed if needed. The background liver was subjected to routine staining methods including hematoxylin-eosin, Masson trichrome, and periodic acid Schiff after diastase. The pathologic differentiation grade of each HCC was assigned via consensus agreement between two experienced pathologists who were blinded to the clinical history and radiologic examination results of patients. According to the HCC pathologic grading system issued in 2010 by the World Health Organization for classification of tumors of the digestive system, HCCs were subcategorized into three grades: WD, MD, and PD. When there were different grades within a tumor, the grade of the tumor was determined by the most predominant differentiation. These procedures were performed as routine examinations. A total of 51 pathologically confirmed HCCs based on surgically resected specimens were included in this study. The pathological classifications of the HCCs included nine WD, 35 MD, and seven PD HCCs.

### 2.3. MRI

MRI signal reception was performed in a 3.0T MRI whole-body scanner (750W, GE Healthcare, Milwaukee, WI, USA) with an eight-element phased array coil. The gradient strength and the gradient slew rate were 50 mTm^−1^ and 200 mTm^−1^ms^−1^, respectively.

The DCE-MRI examination was performed using the tri-directional LAVA (liver acquisition with volume acceleration) protocol with a breath-hold and the following parameters: repetition time/echo time, 4.1 msec/1.9 msec; flip angle, 12°; matrix size, 288×170; section thickness/interslice gap, 4 mm/0 mm; field of view (FOV), 380 mm; rectangle FOV, 0.85. With these parameters, the entire liver can be covered in a single breath-hold of 10 seconds. The DCE-MR images consisted of precontrast, later arterial, portal venous, equilibrium phase, and delay phase images, which were acquired at 0 second, 26 seconds, 60 seconds, 180 seconds, and 300 seconds, respectively, after a rapid injection of 0.1 mmol/kg body weight (0.2 ml/kg) of Gd-DTPA (Magnevist, Bayer-Schering Pharma, Berlin, Germany) at a rate of 2 ml/s. This was immediately followed by a 20 ml saline flush at a rate of 2 ml/s through a power injector.

Other MR sequences were also performed, including in- and opposed-phase spoiled gradient-recalled echo T1-weighted imaging (T1WI), respiratory-triggered T2-weighted fast spin-echo imaging with fat-suppression, and respiratory-triggered single-shot echo-planar DWI with two* b* values (0 and 600 s mm^−1^).

### 2.4. Data Preprocessing

#### 2.4.1. Data Annotation

The generation of a sufficiently large amount of well-annotated training data from existing cases is the key step in the development of the HCC diagnostic model. A tumor location annotation tool was developed based on Python's standard GUI library Thinter, which was used to save the position information of each HCC on Digital Imaging and Communications in Medicine (DICOM) data. After training on five cases that were not included in this study, all annotation was performed manually by one radiologist (X.P. Wang, with 5 years of experience in abdominal imaging) and subsequently verified by the other radiologist (D.W. Yang, with 10 years of experience in abdominal imaging). To minimize bias, both radiologists were blinded to clinical history and pathologic diagnosis. First, a rectangular region of interest (ROI) was placed on the slice with the maximum area of an HCC lesion. The rectangular ROI completely covered the entire lesion as well as a small number of other tissues around the HCC lesion. Second, the numbers of slices at the top and bottom ends of the z-axis were recorded so that the 3D position information about the entire lesion was marked. The 3D position information was used as the input data.

#### 2.4.2. Data Normalization

At the preprocessing stage, we normalized the intensity value of the volume of MR images to the range of [0,1] according to (1). This normalization is helpful for reducing the noise caused by the high intensity values of the images. In contrast to natural images, the intensity values of MR images have a wide range and, accordingly, high resolution, which reflects more details in MR images.

(1) $$\begin{array}{c} I^{\prime }=\frac{\left(I-I_{min}\right)}{\left(I_{max}-I_{min}\right)}, \end{array}$$

where *I*′ and *I* denote the normalized and original intensity values, respectively. *I*_min_ is the minimum intensity value of the whole volume, and *I*_max_ is the maximum intensity value after trimming the top 1% grayscale value. This type of normalization preprocessing has been widely employed in related work [12].

#### 2.4.3. Data Representation with a 4th-Order Tensor

**Figure 1:**
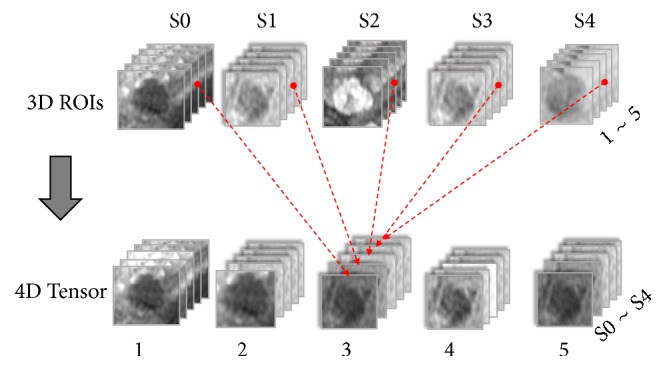
Data representation with a 4th-order tensor.

Based on the location of the HCC obtained at the annotation step, the intensity value of the center slice located at the ROI, that is, the chopped volume enclosing the HCC at each phase, was obtained from the original DICOM data. As the volume of HCC generally varies among different cases, processing by deep neural networks is not feasible. Therefore, the cross-sectional dimension of the HCC was normalized to a fixed size to adapt to the neural network. In this paper, we set the size of the normalized HCC slice as 32×32. Along the vertical angle of view, we selected two neighboring cross-sectional slices at both sides of the center slice with the largest lesion region. Therefore, the size of the HCC lesion volume was set to 32×32×5. Taking into account dynamic information because the HCC lesion has five phases in each case, the data for each HCC lesion were represented as 4th-order tensors with a size of 32×32×5×5. The structure and strategy of the tensor-based representation are illustrated in Figure 1.

In Figure 1, S0 to S4 denote the five phase numbers of the DCE-MR images, and 1 to 5 indicate the cross-sectional slice numbers of each HCC lesion. Based on the tensor-based HCC data representation method, a model for DCE-MR images with 4 dimensions containing 3D spatial and 1D temporal information was established by splicing each 2D slice of the five phases of HCC into a 3rd-order tensor and then splicing the 3rd-order tensors of the five slices into a 4th-order tensor.

#### 2.4.4. Data Augmentation

To realize well-trained deep networks, a large dataset is required to prevent overfitting of the obtained diagnostic model, but existing patient cases are not sufficient. To overcome the shortage of samples, data augmentation was performed referring to the general approach adopted in the computer vision field. In this paper, we performed data augmentation by performing operations of transposition, rotation, and flipping on the samples, that is, the rectangular ROI enclosing the suspicious HCC lesion within MR images, in both the training set and the testing set. After the operations of transposition, 90° rotation, and flipping (horizontal and vertical), the amount of data was increased 8-fold compared with the original dataset.

### 2.5. Deep Learning Model with the MCF-3DCNN

#### 2.5.1. Principle of Model

To reveal the characteristics of the temporal and spatial information of the DCE-MR images to aid HCC diagnosis, we propose a MCF-3DCNN model that contains several separate 3D-CNNs with the same structure. Each independent channel of the 3DCNN deep learning model is employed to extract the features of the 3rd-order tensors of the serial phases of lesion slices at the same cross-sectional layers of HCC MR images. In this way, the local characteristics of the dynamic change and 2D spatial information contributing to lesion diagnosis in the same sectional layer are extracted. This strategy is consistent with clinical diagnosis experience and takes into account both the phasic change and local spatial region information. Furthermore, to provide robust diagnosis, additional neighboring sectional layers are considered. Therefore, by concatenating the output from each channel, the features of each cross-sectional layer reflecting the serial temporal and 2D spatial characteristics are combined to provide comprehensive knowledge of the temporal and 3D spatial information of DCE-MR images of HCC. The concatenated features are then provided to the subsequent category computing network to generate the corresponding diagnosis results. Here, the parameters of the model are trained based on an annotated dataset containing well-labeled HCC lesion regions and grading degrees from real clinical patient cases and experienced radiologists.

#### 2.5.2. Structure of the Model

**Figure 2:**
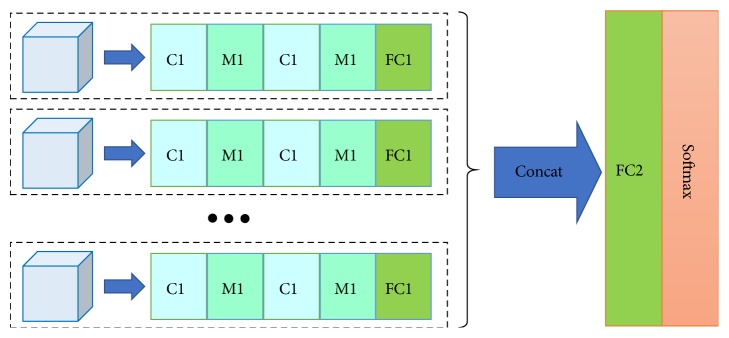
The architecture of the MCF-3DCNN.

Figure 2 illustrates the network structure of the proposed MCF-3DCNN. Our proposed MCF-3DCNN contains five 3D CNNs with the same structure. According to this structure, the 4th-order tensor representation of the data is split into five separate 3rd-order tensors. The 4th dimension in our approach refers to the layer of the cross-section. The remaining 3rd-order tensors contain one temporal dimension and two spatial dimensions, which refer to the phases and the 2D lesion slice at one cross-sectional layer of DCE-MR, respectively. The 3rd-order tensors at the 5 layers are used as the input for the five separate 3D CNNs. Considering the limitation of sample volume, we use a compact structure of the 3D CNN network to avoid the burden of large-scale parameter computing. In the paper, each 3D CNN is configured with two conventional layers denoted as C1 and C2, two max pooling layers denoted as M1 and M2, and one fully connected layer naming FC1. Then, the output of each 3D CNN is concatenated directly as the input of the common fully connected layer FC2. The output of the FC2 layer is connected to the softmax network to compute the category result as a reference for diagnosis.

The fusion structure obviates the need to find a completely novel computing solution to process the four-dimensional tensor. Moreover, the proposed fusion structure facilitates the application of the mature 3DCNN to deal with the four-dimensional data by splitting it into several independent 3D datasets. Therefore, the general experience is referred to in the hyperparameter configuration of each 3DCNN network. First, the basic configuration parameters of the convolution layers, max pooling layers and full connection layers are set in the general way, as listed in Table 2. A rectified linear unit (ReLU) [15] is used as the activation function. The initial values of the convolution kernels are generated under the Gaussian distribution constraint. The adaptive moment estimation (Adam) optimization algorithm [16] is employed in parameter tuning with minimization of cross-entropy loss. Furthermore, to avoid overfitting, learning rate reduction and the dropout method [17] with a ratio of 0.5 are used at the training stage.

### 2.6. Training and Testing Strategy for MCF-3DCNN

As a general problem in medical image analysis, the imbalance of training samples of HCC also needs to be taken into account during the training of our MCF-3DCNN for pathologic grading evaluation. The sample amounts in the different HCC grading categories vary significantly. In fact, the classification performance of trained CNNs based on class-imbalanced samples is prone to fitting the categories with more samples, which is detrimental for obtaining a universal effective model. Various types of solutions have been proposed to overcome the problem of imbalanced samples [18]. In this study, we use a practical compromised label-shuffling method that takes an appropriate number of samples from each category of samples. The determined number is normally the medium sample number of all categories. This is feasible in our application because the balance dataset obtained through resampling from the augmented training data will not significantly influence the results.

For the task of noninvasive assessment of the pathologic grade of HCC in this study, the collected HCC samples labeled with three degrees of differentiation was reorganized into three categories. After data augmentation, the training dataset was resampled using a compromise label-shuffling method. Specifically, we randomly resampled 128 samples from each category in the augmented training set, and repeated extraction was allowed during resampling. The test dataset was subjected to similar processing with a sample number setting of 64. The specifications of the sample numbers in the training and testing datasets are listed in Table 3, where each set of three numbers, such as “4 | 32 | 128”, indicates the sample numbers in the three categories of original collection, data augmentation, and resampling with the compromise label-shuffling method, respectively.

The MCF-3D CNN model in this study was established using Keras (https://keras.io/) and TensorFlow [19]. The dataset was formed based on the expert annotation and subsequent data preprocessing, augmentation and resampling. Then, the MCF-3D CNN was trained on the training set with a batch size and epoch of 32 and 1000, respectively. After the MCF-3DCNN model was trained, the diagnostic performance of noninvasive differentiation of the pathologic grade of HCC was evaluated based on the testing sets.

### 2.7. Statistical Analysis

For statistical analyses, the Scikit-learn toolkit version 0.19.1 (http://scikit-learn.org/stable/ index.html) was used. The average accuracy, as well as the recall and precision, was generally calculated among ten test sessions of the evaluation of the pathologic grade of HCC by using the MCF-3DCNN. Data were expressed as the mean ± standard deviation. The Matplotlib toolkit version 2.2.2 (https://matplotlib.org/) was used to map the confusion matrix and perform the receiver operating characteristic (ROC) analyses. The sensitivity, specificity, accuracy, and area under the receiver operating characteristic curve (AUC) were calculated to evaluate the performance of the MCF-3DCNN in discriminating WD HCCs, MD HCCs, and PD HCCs from other cases using the test data.

## 3. Results

### 3.1. MRI Appearance of HCCs

Our study did not consider the proliferative properties of cirrhotic nodules, including regenerative nodules and dysplastic nodules. The aim of this study was to differentiate pathologic grades of HCCs. Moreover, the pathologic reports of the surgically resected HCC specimens used in our retrospective study rarely mentioned the details of associated cirrhotic nodules.

Fifty-one pathologically confirmed HCCs from 42 patients were included in this study. Of the 42 patients, three had two lesions, and three had three lesions. Thirty-five of 51 HCCs presented in the right lobe, and the remaining 16 tumors were located in the left lobe. The average diameters of the lesions in different groups varied, with lesions of 2.83±1.78 cm, 3.45±1.64 cm, and 2.75±0.99 cm in the WD, MD, and PD HCC groups, respectively. However, the pathologic differentiation of HCCs cannot be simply determined based on tumor size.

**Figure 3:**
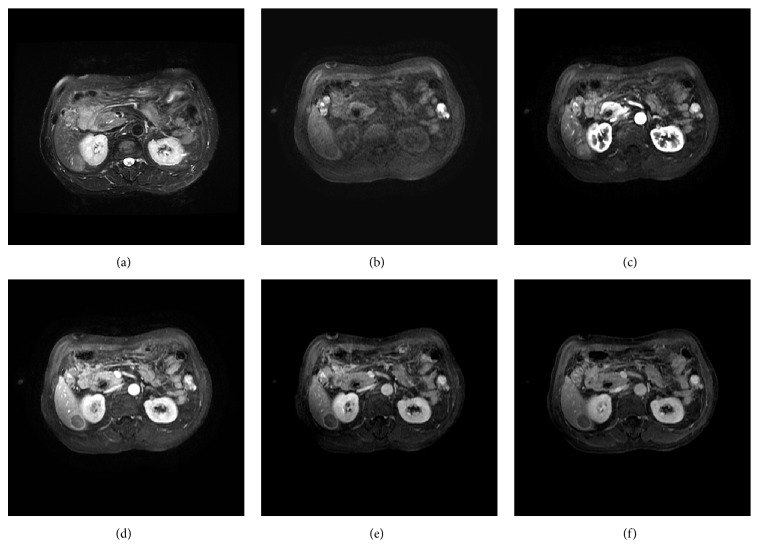
Axial MR images and pathologic image of a 70-year-old man with HCC. (a) A fat-suppressed T2-weighted fast spin-echo image shows an oval-shaped, slightly hyperintense neoplasm in the dorsal part of segment VI, with a maximum diameter of 2.6 cm. Axial precontrast (b), late artery phase (c), portal vein phase (d), equilibrium phase (e), and delay phase (f) T1-weighted 3D GRE images demonstrate a hypointense appearance of the lesion on precontrast T1-weighted images (b), high enhancement in the late arterial phase, (c) and washout in the portal vein phase (d) with an enhancing capsule that can clearly be observed in the equilibrium phase (e) and delay phase (f); all of these MRI features are consistent with typical HCC. The tumor was successfully surgically resected and was pathologically confirmed as a WD HCC.

**Figure 4:**
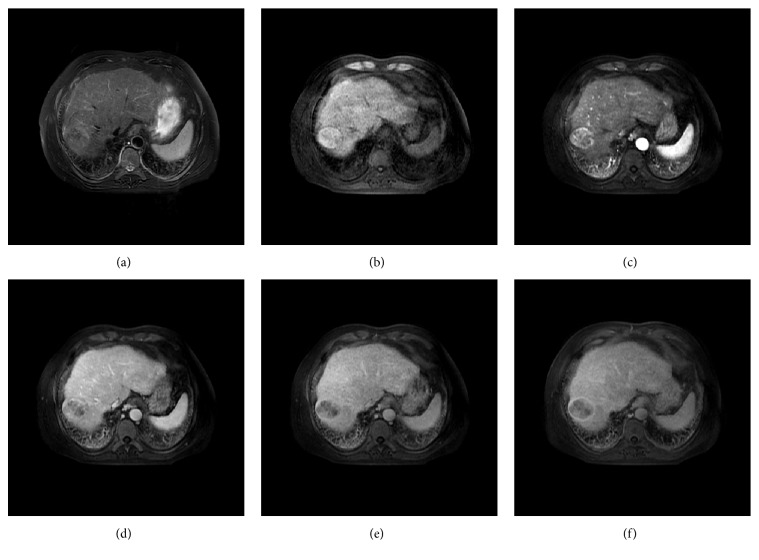
Axial MR images and pathologic image of a 52-year-old man with HCC. (a) A fat-suppressed T2-weighted fast spin-echo image shows an oval-shaped, heterogeneous, slightly hyperintense neoplasm in segment VII with a maximum diameter of 4.6 cm. Axial precontrast (b), late artery phase (c), portal vein phase (d), equilibrium phase (e), and delay phase (f) T1-weighted 3D GRE images show a hyperintense appearance of the lesion on precontrast T1-weighted images (b), obvious enhancement in the late arterial phase (c), and washout in the portal vein phase (d); an enhancing capsule was detectable in the equilibrium phase (e) and was more obvious in the delay phase (f); all of these MRI features are consistent with typical HCC. The tumor was successfully surgically resected and was pathologically confirmed as an MD HCC with no vascular invasion.

**Figure 5:**
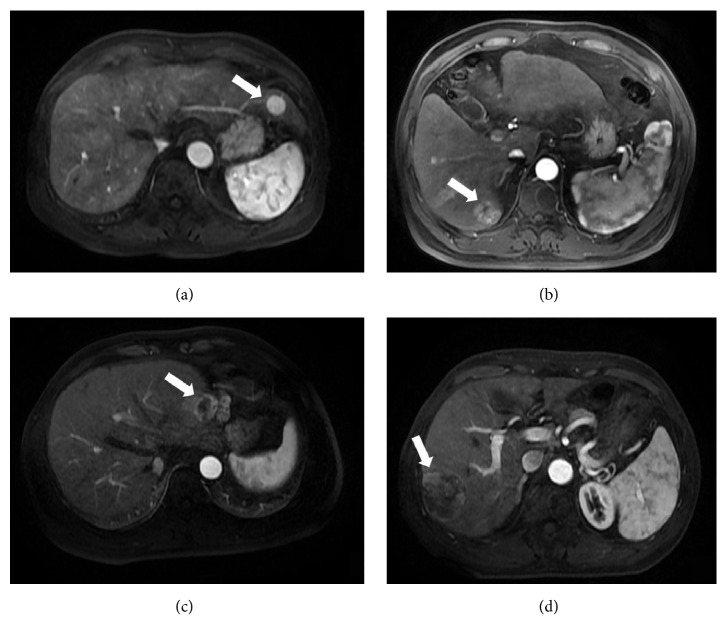
Axial late arterial phase DCE-MR images of four pathologically confirmed PD HCCs. The imaging appearances in the arterial phase of different PD HCCs varied, presenting with homogeneous hyperenhancement (a), heterogeneous hyperenhancement with a small area of necrosis (b), heterogeneous hyperenhancement with a large area of necrosis (c), and hyperenhancement in some parts of tumor (d).

More than half of the HCCs (29/51) exhibited the characteristic MRI features of HCC, including a moderate hyperintense appearance on T2-weighted imaging (T2WI), a hypointense appearance on T1WI, arterial phase hyperenhancement, and washout in the portal vein or delay phase. Additionally, an enhancing capsule appeared in the equilibrium phase or delay phase (Figure 3). However, some uncommon appearances were also observed, such as a hypo- or isointense appearance on T2WI (7/51 lesions), a hyper- or isointense appearance on T1WI (10/51 lesions) (Figure 4), the absence of arterial phase hyperenhancement (3/51 lesions), and an unclear enhancing capsule (4/51 lesions). In general, the various imaging features of HCCs were independent of the lesion size, and the imaging appearances of different PD HCCs on DCE-MR images varied significantly (Figure 5).

### 3.2. Computer-Aided Diagnosis of HCCs

To ensure the reliability of the results, we repeated the experiment 10 times and then calculated the averages and standard deviations of all parameters obtained from the experiments. The average accuracy of the gross differentiation of the pathologic grade of HCC via the MCF-3DCNN in the test data was 0.7396±0.0104, and the average sensitivity and precision were 0.7396±0.0104 and 0.8042±0.0198, respectively.

**Figure 6:**
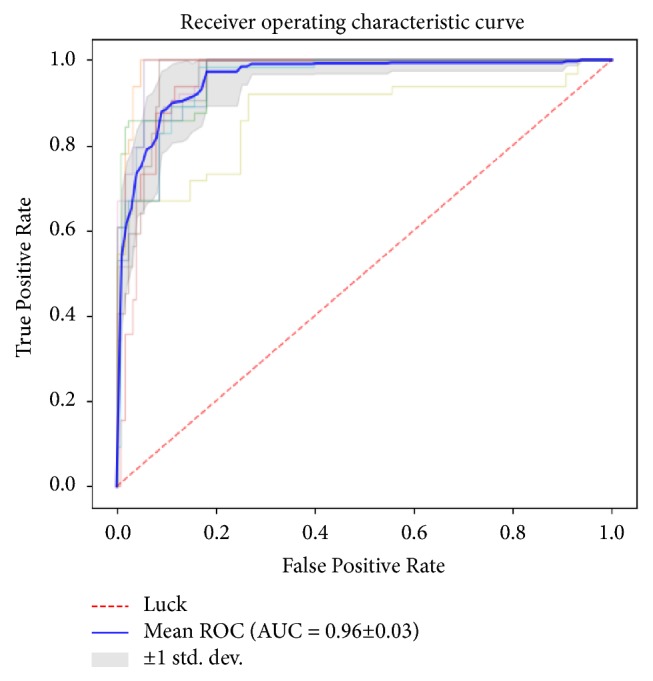
The average area under the ROC curve for 3DCNN for discriminating WD HCCs from the others was 0.96.

**Figure 7:**
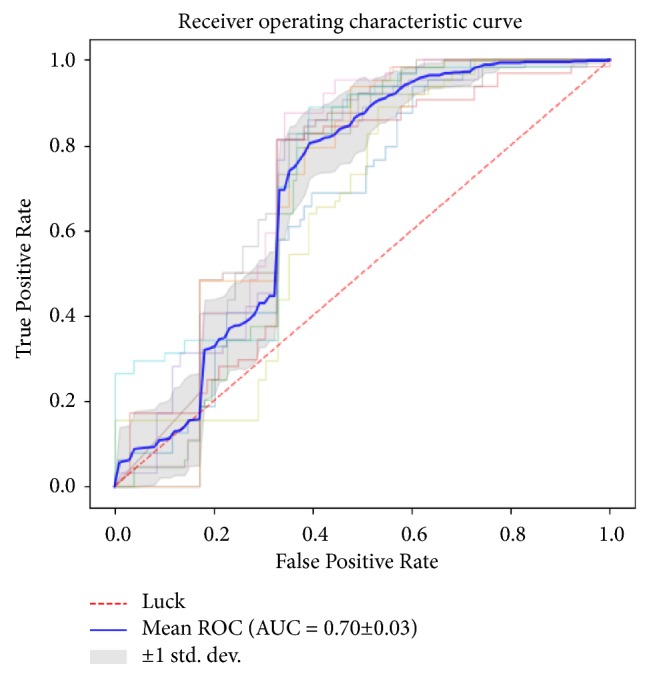
The average area under the ROC curve for 3DCNN for discriminating MD HCCs from the others was 0.71.

**Figure 8:**
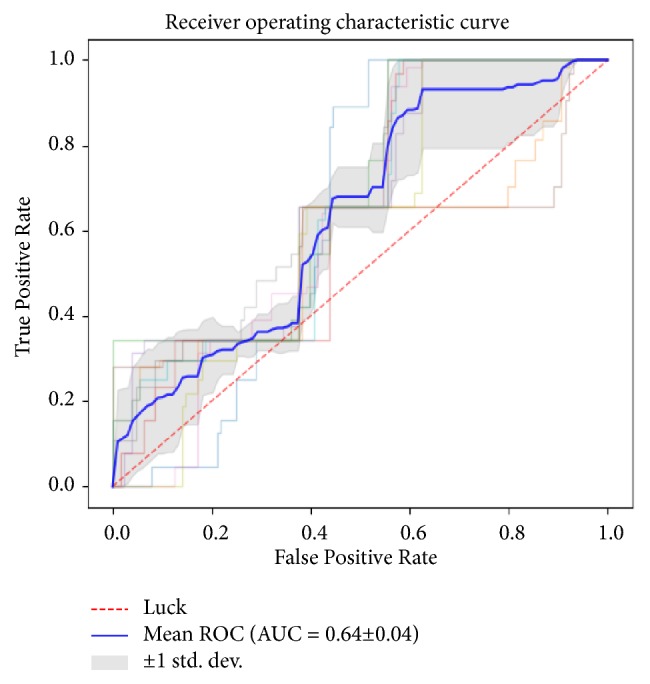
The average area under the ROC curve for 3DCNN for discriminating PD HCCs from the others was 0.64.

The diagnostic performance of the MCF-3DCNN in differentiating a specific, single type of pathologic grade of HCC from the others in the test data is shown in Table 4. The MCF-3DCNN achieved the highest diagnostic performance in discriminating WD HCCs from the others, with an average AUC, accuracy, sensitivity, and specificity of 0.96, 91.00%, 96.88%, and 89.62%, respectively (Figure 6). However, the diagnostic performance of the MCF-3DCNN in discriminating MD (Figure 7) and PD HCCs (Figure 8) from the others was relatively low, with average AUCs of 0.71 and 0.64, respectively.

### 3.3. Time of Processing for Each Step

We calculated the time consumed by the training and testing processes. The experiments were performed using an Ubuntu16.04 LTS operating system with a GeForce GTX 1080 (NVIDIA, Santa Clara, Calif) graphics processing unit, a Core i7-6700K 4.00-GHz×8 (Intel, Santa Clara, Calif) central processing unit, and 16 GB of random access memory. Approximately 916 seconds were required to independently train our model ten times. In each independent experiment, 0.5082±0.2743 seconds were required to evaluate all 192 samples.

We integrated the well-trained model into a decision support system and selected the tumor region from the whole abdominal MR image manually. The selected tumor area was extracted from the original DICOM data, and then the prediction scores of the region were calculated using the trained model. The entire process required approximately 3.2131±0.0864 seconds per sample, not including the time required to manually select the tumor region. Of the total time spent, only approximately 0.0352±0.0072 seconds were required for prediction; most of the time was spent on preprocessing of the DICOM data and extraction of tumor data from the DCE-MRI data.

## 4. Discussion

We investigated whether different pathologic grades of HCC could be differentiated in DCE-MR images using deep learning with 3DCNN models. Our study indicated that the MCF-3DCNN showed a high diagnostic accuracy rate of 0.7396±0.0104 in the general evaluation of the pathologic grades of HCC.

HCC is generally considered a hypervascular tumor from the perspective of angiography [20]. The number of portal tracts is significantly reduced in HCC, and the number of intratumoral arterioles increase as the tumor becomes increasingly dedifferentiated. The changes in the hemodynamics of HCC correlate well with its pathologic grade. The correlation between the enhancement pattern on dynamic MR images and pathologic grades was validated in a study by Okamoto D et al. [21], which found that tumors with worse pathologic grades usually showed an earlier washout pattern. Therefore, this study selected DCE-MR images with five phases for the texture-based analysis to directly characterize the close relationship of structural morphology with the pathologic grade of HCCs.

In this study, we proposed using modified deep learning networks with multiple-channel fusing of several 3D CNNs for noninvasive evaluation of the pathologic grade of HCC. The proposed MCF-3D CNN model is beneficial for analyzing DCE-MR images and approximates clinical experience by taking into account the dynamic change in serial phases and the morphological information in different scanning slices. To our knowledge, few studies have applied the CNN method to assess the pathologic grade of HCC. Some studies [7, 22] have used traditional machine learning to diagnose liver masses by placing regions of interest on tumors and extracting features such as quantitative texture parameters. Wu Z et al. [7] reported a study that used conventional analysis to characterize the malignancy of HCC based on arterial phase images. The authors found that compared with high-grade HCCs, low-grade HCCs showed an increase in mean intensity and a decrease in gray-level nonuniformity (GLN) in four directions. In addition, the AUC values of the average intensity and GLN in four directions were 0.918, 0.846, 0.836, 0.827, and 0.838, respectively. However, this was only a relevant study that studied the relationship between pathologic grade and two selected parameters. More importantly, the method used in this study could not fully extract and utilize the timing and space characteristics contained in the multiphase DCE-MR images, because only two selective parameters (GLN and mean intensity) extracted from single arterial phase images were employed in the study.

The deep learning method has been widely applied in the diagnosis and staging of liver disease. Yasaka K et al. [23] used the deep learning method of CNNs to characterize liver tumors based on DCE computed tomography (CT). The authors reported that deep learning with CNN performed well diagnostically in the differentiation of liver masses using dynamic CT, with a median accuracy of 0.84. Yasaka K et al. [24] also reported that the CNN model exhibited a high diagnostic performance in the staging of liver fibrosis. However, unlike our study, their studies used only one single-section JPEG image that was converted from DICOM images of each lesion rather than intact DICOM images of entire focal lesions; this approach might have caused information loss and might have resulted in the relatively low sensitivity and accuracy observed in differentiation. Because the texture characteristics of HCC were comprehensively captured from DICOM images of all of the sections, the results of our study were more accurate.

Moreover, compared to two-dimensional CNN, three-dimensional CNN has gained more attention with respect to the action recognition of the video, which takes into account both spatial and temporal information [25]. Therefore, deep learning with 3D CNNs enables all of the information contained in the three-dimensional space to be used, while only the local deep spatial-temporal features are learned in conventional machine learning. Therefore, our method has the potential to evaluate the pathologic grade of HCC based on DCE-MRI with obvious spatial and temporal characteristics and does not depend on the radiologist's experience level.

The diagnostic performance of this method when discriminating different pathological grades of HCC was variable; specifically, the best diagnostic ability was observed in WD HCC (AUC=0.96). This finding is superior, to some extent, to that of published studies that used MRI texture analysis. Wu M et al. [22] reported that a radiomics analysis based T1WI and T2WI could potentially distinguish high-grade and low-grade HCCs. However, the diagnostic performances, which were reflected as the AUCs of radiomics and a combination of radiomics and clinical factors, were approximately 0.742 and 0.800, respectively. Based on Gd-DTPA-enhanced MR images that were similar to our input data, the maximal AUC in the study by Wu Z et al. [7] using texture analysis for the characterization of low-grade HCCs was 0.918. Considering that the low-grade HCCs according to the Edmondson-Steiner (E-S) system included in prior studies could be regarded as WD HCCs [26, 27], we found that our results were slightly better. While more investigations are needed, two possible reasons may contribute to the difference in diagnostic performance between our study and previous studies: the priority of DCE-MR images over either T1WI or T2WI in the characterization of pathologic grades and the potential advantage of the deep learning method over the radiomics method in the extraction features.

Our diagnostic model showed poor performances in discriminating MD (AUC=0.71) and PD (AUC=0.64) HCCs. Several explanations could exist for this relatively poor performance. First, PD and MD HCCs exhibit diverse characteristics in terms of cell structure, vascular infiltration, mesenchymal abundance, necrosis and portal vein thrombosis compared to WD HCC [28], leading to more diversified appearances on DCE-MR images, as partly shown in Figure 5. As a result, it was more difficult for the MCF-3DCNN model to clearly learn the intrinsic texture features of PD and MD HCCs. Moreover, the relatively small size of the samples hampered the diagnostic performance of the method. Several studies have investigated the effectiveness of some traditional MRI techniques in the discrimination of MD or PD HCCs, and the results varied. Huang X et al. [29] found that a specific contrast-to-noise ratio in the hepatobiliary phase of gadobenate dimeglumine-enhanced MRI had the potential to distinguish MD HCCs from PD HCCs with a sensitivity and specificity of 84.6% and 60.0%. Ogihara Y et al. [30] reported that parameters derived from DWI exhibited good performances for discrimination of PD HCCs, with AUCs in the range of 0.711 to 0.864. Ichikawa S et al. [31] concluded that the parameters of intravoxel incoherent motion such as D, D^*∗*^, and f exhibited variable discrimination abilities with different fitting methods, with AUCs ranging from 0.463 to 0.881. The relatively poor discrimination ability of our model requires future improvements.

There are several limitations of our study. First, the sample size was relatively small, and the distribution of patients among the three pathologic grades was imbalanced. This study used methods to reduce the impact of the imbalance problem and augmented the data to increase the data scale, but future studies should add many cases reflecting the intrinsic characteristics of HCC lesions. Second, the accuracy when differentiating MD or PD HCC was not as good as that for WD HCC. A solution for extracting discriminative features from the diverse appearance of HCC should be proposed in a future study. Third, we have not yet built a model that can automatically detect HCC. The detection of HCCs, especially small HCCs, is the first step in the clinical procedure of diagnosis, staging, and treatment. Because the CNN model was found to be effective for the differentiation of HCC from cirrhotic background, we intend to conduct this study in the future.

In conclusion, this pilot study indicated that the MCF-3DCNN model may be valuable for the noninvasive evaluation of the pathologic grade of HCCs; however, further improvement would be necessary to achieve a better diagnostic performance for MD and PD HCCs.

## Figures and Tables

**Table 1:** Clinical characteristics of 42 patients and pathological features of the tumors.

	Well-differentiated	Moderately differentiated	Poorly differentiated
Number	9	35	7
Age (years)	61.44±6.80	58.31±7.70	61.857±11.00
Sex			
Male	7	30	6
Female	2	5	1
Tumor Diameter (mm)			
Mean	2.83±1.78	3.45±1.64	2.75±0.99
AFP			
Mean	44.75±102.59	54.88±84.46	1350.76±1545.56
Cirrhosis	7(77.8%)	18(51.4%)	5(71.4%)
Child-Pugh Score			
5	4(44.4%)	24(68.6%)	5(71.4%)
6	4(44.4%)	6(17.1%)	1(14.3%)
7	1(11.1%)	5(14.3%)	1(14.3%)
Child-Pugh stage			
A	8(88.9%)	30(85.7%)	6(85.7%)
B	1(11.1%)	5(14.3%)	1(14.3%)
BCLC Classification			
A	3(33.3%)	26(74.3%)	4(57.1%)
B	6(66.7%)	9(25.7%)	0(0%)
C	0(0%)	0(0%)	3(42.9%)

**Table 2:** Specification of the basic parameters of the MCF-3D CNN.

Layer	Input Size	Kernel Size	Kernel Number	Stride	Output Size
C1	32×32×5	3×3×3	6	1×1×1	30×30×3
M1	30×30×3	2×2×1	6	2×2×1	15×15×3
C2	15×15×3	4×4×3	8	1×1×1	12×12×1
M2	12×12×1	2×2×1	8	2	6×6×1
FC1	36	-	32	-	32
FC2	160	-	32	-	32
Softmax	32	-	3	-	3

**Table 3:** Specification of the numbers of samples in the three categories of the dataset for collection/augmentation/resampling.

Datasets	Number of HCC samples | Augmentation | Label Shuffling		
	Poorly	Moderately	Well
Training	4 | 32 | *128*	26 | 208 | *128*	6 | 48 | *128*
Testing	3 | 24 | *64*	9 | 72 | *64*	3 | 24 | *64*
Total	7 | 72 | *192*	35 | 280 | *192*	9 | 56 | *192*

**Table 4:** The diagnostic performance of each pathologic grade of HCC using test data.

Data	Accuracy	Sensitivity	Specificity	AUC
*Poorly*	0.7708±0.0539	0.3437±0.0449	0.9429±0.0612	0.6448±0.0469
*Moderately*	0.6823±0.0221	0.7031±0.0453	0.6719±0.0341	0.7067±0.0322
*Well*	0.9100±0.0303	0.9688±0.0403	0.8962±0.0593	0.9641±0.0391

*∗*Data expressed as the mean±SD.

## Data Availability

The DICOM data used to support the findings of this study are related to patient privacy, which may be released upon application to the Department of Radiology, Beijing Friendship Hospital, Capital Medical University, China, who can be contacted at Dawei-yang@vip.163.com.
